# Reliability of Concussion Signs and Symptoms Reporting Among Former Professional American-Style Football Players

**DOI:** 10.1177/08977151251362274

**Published:** 2025-07-22

**Authors:** Niki A. Konstantinides, Rachel Grashow, Heather DiGregorio, Elizabeth Nolan, Frank E. Speizer, Aaron L. Baggish, Ross D. Zafonte, Marc G. Weisskopf

**Affiliations:** ^1^ Harvard Medical School, Football Players Health Study at Harvard University, Boston, Massachusetts, USA.; ^2^ Department of Environmental Health, Harvard T.H. Chan School of Public Health, Boston, Massachusetts, USA.; ^3^ Channing Division of Network Medicine, Brigham and Women’s Hospital, Harvard Medical School, Boston, Massachusetts, USA.; ^4^ Cardiovascular Performance Program, Massachusetts General Hospital, Boston, Massachusetts, USA.; ^5^ Department of Cardiology, Lausanne University Hospital (CHUV) and Institute for Sport Science, University of Lausanne (ISSUL), Lausanne, Switzerland.; ^6^ Department of Physical Medicine and Rehabilitation, Spaulding Rehabilitation Hospital, Charlestown, Massachusetts, USA.; ^7^ Department of Physical Medicine and Rehabilitation and Anesthesiology, University of Missouri, Columbia, Missouri, USA.

**Keywords:** concussion, football, traumatic brain injury

## Abstract

Retrospective evaluations of repeated head injury are needed to better understand associations between head injury exposure and later-life deleterious outcomes. However, there is limited assessment of whether head injury recall assessments produce consistent measures over time, and no assessment of whether the reporting is related to current health status. The concussion signs and symptoms scale (CSS; developed for the Football Players Health Study at Harvard University) was designed to measure cumulative head injury exposure history by asking about the frequency of 10 CSS during active football play. Responses are summed with a total CSS range of 0–130. Former professional American-style football players completed the CSS at two timepoints. A subset of participants also reported on current health (subjective cognitive symptoms [Quality of Life in Neurological Disorders], depression [Patient Health Questionnaire], anxiety [Generalized Anxiety Disorder], pain [Patient-Reported Outcome Measurement Information System (PROMIS) Global], and overall health [PROMIS Global]) at each timepoint. To examine reporting consistency and recall bias, we calculated the Spearman correlation between measures assessed an average of 74.5 (standard deviation [SD] = 41.2) months apart and estimated associations between change in demographic, football-related, and current health factors and change in CSS (ΔCSS) over time using multivariable linear regression. Across the 335 participants, the mean (SD) CSS score at times 1 and 2 were 30.2 (25.5) and 29.1 (25.2), respectively, with an average change in CSS (ΔCSS) of −1.1 (SD = 19.8). There was no significant association between ΔCSS and years since play, months between timepoints, or age at time 1 (0.49 < *p* < 0.84). Eighty-one (24.2%) participants completed identical questions on current health factors at times 1 and 2. In separate multivariable models, there was no association between changes in pain, cognitive symptoms, health, depression, and anxiety reporting and ΔCSS (0.17 < *p* < 0.92). On average, the CSS score as a measure of retrospective concussion exposure did not change meaningfully over an average of 75 months, and changes in current health status were not significantly associated with ΔCSS. Results suggest that the CSS scale is stable over time and appears robust against changes in health status. The CSS should be considered for other retrospective studies of brain-injured populations to measure prior cumulative concussion history.

## Introduction

Previous exposure to head injury has been associated with a number of later-life deleterious outcomes in former professional American-style football (ASF) players.^1–4^ Understanding the long-term health impacts of repetitive head injury requires tools that accurately and reliably capture prior head injury exposure. Retrospective assessments of such injuries have been historically difficult to quantify because (a) there is no preestablished definition of concussion^5^; (b) injuries may have occurred decades prior to the health assessment; (c) culture and diagnostics around concussion have changed over time^5^; (d) equipment and football safety rules have changed^6,7^; and (e) data from helmet^8^ or mouthpiece accelerometers that has been paired with injury data still require further research and validation^9,10^ and cannot be applied where such devices were not in place.

There are few methods currently used to capture cumulative concussion history. For the general population, researchers have previously used the number of head injuries with loss of consciousness (LOC) due to it being a more easily recalled exposure and indicator of more severe traumatic brain injury (TBI).^11–13^ However, relying on the history of LOC may greatly underestimate reported numbers of concussion, as fewer than 10% of sports concussions have been estimated to involve LOC.^14^ The use of the Ohio State University Traumatic Brain Injury Identification Method (OSU TBI-ID),^15^ an administered survey in which a trained interviewer asks questions of participants to ascertain their lifetime history of TBI with and without LOC, has recently become more common in retrospective TBI studies.^15,16^ However, due to the interview design of the OSU TBI-ID, it can only be administered in-person and may be difficult to use in large cohorts who may be geographically dispersed. Although recently, the OSU TBI-ID has shown some indices to be reliable with interviews taking place over the phone.^17^ In military personnel exposed to repetitive head injury, the Blast Exposure Threshold Survey, a self-report survey, was recently designed to measure lifetime blast exposure^18^ and was found to have convergent and discriminant validity, with high scores also associated with worse neurobehavioral outcomes after mild TBI.^19^ To measure cumulative repetitive head injury in former professional ASF players, the Football Players Health Study (FPHS) at Harvard University designed the concussion signs and symptoms (CSS) scale. Unlike the OSU TBI-ID, the CSS scale is administered electronically to participants and completed without assistance. Both the CSS scale and the OSU TBI-ID avoid the use of leading terms (e.g., “head injury,” “TBI,” “concussion,” “knocked out”) as these may be interpreted differently. Instead, the CSS scale elicits recall of injuries by querying “a blow to the head, neck, or body” and asking about subsequent symptoms.^15^ For these occurrences, symptoms that historically have been associated with concussions^20,21^ are summed to create a CSS score. Higher scores on the CSS have been associated with outcomes commonly associated with head injury, such as depression,^4^ anxiety,^4^ self-reported cognitive dysfunction,^4^ endocrine dysfunction,^1^ and cardiometabolic outcomes.^2,3^

Recall bias poses a potential limitation for retrospective recall assessments such as the OSU TBI-ID and CSS in former athletes,^22^ if recall depended on current health status. Media attention highlighting the association between concussion history and mental health or cognitive symptoms^23–25^ may contribute to recall bias, as those currently experiencing mental health challenges^26^ or cognitive difficulties^26^ may be more likely to overreport concussion signs, symptoms, or characteristics, compared to those without such issues. Thus, it is important to determine whether these kinds of assessments are stable over time and, importantly, whether current health conditions affect the reporting. The current report describes the findings of the first test–retest reliability study of the CSS scale using a subset of participants from a larger cohort study. We additionally investigated associations between changes in health factors and differences in reporting on the CSS scale at times 1 and 2 (average ∼6.1 years apart) to assess whether current health conditions affect CSS reporting.

## Methods

### Participant recruitment

The FPHS at Harvard University^27^ enrolled former professional ASF players who contracted with a professional football league (e.g., the National Football League or NFL) after 1960 when hard plastic helmets were formally adopted.^28^ Using residential mail and email addresses obtained from the NFL Players Association 18,065 former players were invited to participate in either a hard copy or online questionnaire (initial survey), out of which 4509 (25%) enrolled by completing the baseline survey. From the original cohort, a subsample of 335 participants provided complete information on concussion exposure at two timepoints, of which 81 (24.18%) participants also completed current health status surveys twice (Supplementary Table S1). This study was approved by the Institutional Review Board of the Harvard T.H. Chan School of Public Health, and participants provided informed consent prior to enrollment.

### Demographics measures

The baseline survey included questions pertaining to age and self-identified race (Black/African American, White/Caucasian, American Indian/Alaskan Native/Native Hawaiian/Pacific Islander/Asian/other, and missing). Race was subsequently categorized into Black, White, Latino/Asian/Native American/Pacific Islander due to the historical rates of participation for these groups in ASF, and missing.^29^

### Head injury assessment

The CSS scale was developed for the FPHS to assess concussion history while having actively played football and uses 10 items drawn from the validated postconcussion scale (Lovell and Collins^21^; Chen et al.^30^) that measures acute symptoms following a head injury. Specifically, participants were asked, “While playing or practicing football, did you experience a blow to the head, neck, or upper body followed by:…” with options as follows: headaches, nausea, dizziness, LOC, memory problems, disorientation, confusion, seizure, visual problems, and feeling unsteady on your feet. Participants then selected how many times each of these symptoms occurred by selecting from the following: no, once, 2–5 times, 6–10 times, 11 or more. Scores were categorized and quantified as no (0), once (1), 2–5 (3.5), 6–10 (8), 11 or more (13), and summed resulting in a scale that could range from 0 to 130. The CSS summary score at time 1 (CSS_1_) was subtracted from that at time 2 (CSS_2_) to calculate the CSS difference score (ΔCSS = CSS_2_ − CSS_1_) for all participants. If a participant was missing any symptom score at time 1 or 2, they were removed from the analysis.

### Additional football exposures

Participants provided the first and last calendar year they played professional football, which were used to calculate career duration. We determined years since play using the year of the survey completion and the last calendar year of play. Participants were also asked, “During your professional football career, what position(s) did you most often play? *(Mark all that apply)*” and offered the following categories: offensive line, defensive line, linebacker, defensive back, running back, wide receiver, tight end, quarterback, kicker/punter, special teams. These positions were classified into a categorical lineman status variable, whereby offensive line and defensive line were considered linemen, and linebacker, defensive back, running back, wide receiver, tight end, quarterback, kicker/punter, and special teams were considered nonlinemen.

### Current health measures

Subjective cognitive symptoms were measured using the Quality of Life in Neurological Disorders (Neuro-QOL), a validated and standardized quality of life assessment applicable across neurological conditions.^31,32^ The Neuro-QOL produces raw scores that are converted into normative T-scores (mean [SD] 50 [10]) based on US general population norms, with low scores indicating more cognitive symptoms. Given that other scales in this study associate worse functioning with higher scores, Neuro-QOL T-scores were inverted such that higher scores reflected worse cognitive symptoms.

Pain and health domains of the Patient-Reported Outcome Measurement Information System Global Health scale^33–36^ were also included. Pain intensity was assessed with the question, “In the past 7 days, how would you rate your pain on average?” Zero represented no pain, and 10 represented the worse imaginable pain for the participant. Overall health was determined using the question, “In general, would you say your health is:…” with options: 5 = excellent, 4 = very good, 3 = good, 2 = fair, 1 = poor.^36^ Final scores were then recoded such that higher scores reflected worse overall health to reflect the scores of all other scales used in this study. To assess depression and anxiety symptom severities over the past two weeks, the two-item Patient Health Questionnaire (PHQ-2; Löwe, Kroenke, and Gräfe 2005^37^) and two-item Generalized Anxiety Disorder (GAD-2; Delgadillo et al. 2012^38^), respectively, were used. Both the PHQ-2 and GAD-2 response options include not at all, several days, more than half the days, and nearly every day. If one PHQ-2 or GAD-2 response was missing, it was assumed to be “not at all” if the other was answered.

### Statistical analysis

A total of 412 participants completed the CSS at time 1 and time 2; 77 (18.7%) had at least one missing symptom reported and were removed from analysis. Exclusion of these participants did not alter the overall characteristics of the total population studied (Supplementary Table S2); however, participants who had incomplete CSS responses were on average older than those who had complete CSS responses (Supplementary Table S2).

We calculated the Spearman’s rank correlation between CSS scores across the two time points. We used linear regression to assess whether demographics and survey-related measures (age, race, lineman status, months between survey completion, and years since play) predicted ΔCSS. We then considered the association between change in health measures between time 1 and time 2 (Neuro-QOL, pain severity, poor health, depression, anxiety) and ΔCSS as the outcome in separate models for each health outcome. These models were adjusted for years since play, lineman status, race, and age. Statistical significance was considered at *p* < 0.05, and all analyses were conducted using R Language for Statistical Computing.^39^

## Results

**FIG. 1.**
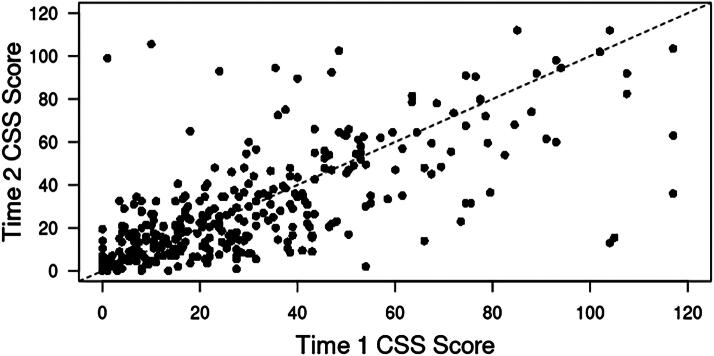
Scatterplot displaying time CSS_1_ versus CSS_2_ score. The black dashed line represents the identity line. CSS, concussion signs and symptoms scale.

Among the 335 participants who completed the CSS at times 1 and 2, average (SD) age was 52.1 (14.2) years, and 114 (34.3%) participants self-identified as Black. The mean (SD) number of years since play was 24.5 (13.7), with a majority having played as a nonlineman 216 (64.5%). On average, 74.5 (SD = 41.2) months elapsed between CSS completion at time 1 and time 2. The average (SD) CSS scores at time 1 and time 2 were 30.2 (25.6) and 29.1 (25.3), respectively. The average CSS difference score was −1.0 (19.9) (Table 1). The correlation between scores at the two times was (rho = 0.70) (Fig. 1). In a multivariable model, there were no significant associations between demographic or football characteristics and ΔCSS, and even the point estimates reflected very small differences relative to the distribution of CSS scores (Table 2).

**Table 1.** Demographics of All Participants with Two Concussion Signs and Symptoms Scale Scores

	Total (*N* = 335)
Age, mean (SD)	52.1 (14.2)
Race	
Black	115 (34.3%)
White	205 (61.2%)
Other	9 (2.7%)
Missing	6 (1.8%)
Years since play, mean (SD)	24.5 (13.7)
Lineman status	
Yes	119 (35.5%)
Months between survey completion, mean (SD)	74.5 (41.2)
Missing, *N*	3
CSS score at time 1, mean (SD)	30.2 (25.5)
CSS score at time 2, mean (SD)	29.1 (25.2)
CSS score difference, mean (SD)	−1.1 (19.8)

CSS, concussion signs and symptoms scale; SD, standard deviation.

**Table 2.** Effect Estimates, 95% Confidence Intervals, and *p*-Values for the Multivariable Model with Predictors: Months Between Survey Completion, Years Since Play, and Age, on ΔCSS

Variable	Effect estimate	95% CI	*p*
Months between survey completion	−0.01	−0.07, 0.04	0.61
Years since play	0.43	−0.23, 1.08	0.20
Years of age	−0.37	−1.01, 0.27	0.26
Lineman status	−1.37	−5.97, 3.23	0.56
Race^a^			
White	0.28	−4.54, 5.10	0.91
Other	8.71	−4.90, 22.33	0.21
Missing	−8.86	−25.48, 7.77	0.30

^a^ Black players are the reference group.

CI, confidence interval; CSS, concussion signs and symptoms scale.

The 81 (24.2%) participants who completed identical questions on current health factors at times 1 and 2 were on average older (mean age ± SD 59.0 ± 13.2) than the full study population, and 34 (42%) self-identified as Black (Supplementary Table S3). Similar to the original cohort, a majority played as a nonlineman (52; 64.2%) and played on average 30.4 ± 13.4 years ago (Supplementary Table S3). Mean CSS scores at time 1 and time 2 were essentially identical in this group (mean ± SD ΔCSS = 0.2 ± 12.3). In no case did change in a health outcome from time 1 to time 2 predict change in the CSS score (Table 3), and most point estimates were even in the direction of lower CSS reporting with worse health reporting. When models were run without adjusting for demographic and football-related factors, no significant changes in point estimates were observed (results not shown).

**Table 3.** Change in CSS Score from Time 1 to Time 2, 95% Confidence Intervals, and *p*-Values, per Unit Worse Health Outcome Scale from Time 1 to Time 2 for Different Health Outcomes

Health outcome	Effect estimate	95% CI	*p*
Pain	−1.41	−4.07, 1.25	0.30
Perceived cognitive difficulties	0.06	−1.10, 1.22	0.92
General health	−2.36	−8.90, 4.14	0.47
Depression	−2.36	−5.71, 1.00	0.17
Anxiety	−1.31	−4.74, 2.12	0.45

CI, confidence interval; CSS, concussion signs and symptoms scale.

## Discussion

The results of this study support the test–retest reliability of a specifically developed scale that retrospectively measures cumulative football-related exposure to head injury among a population of former professional ASF players. We found a strong correlation (0.7) between CSS scores reported about 75 months (6.25 years) apart, and there was little difference in total CSS score reported at the two times: on average, 1 point lower at time 2 out of a range of 130. Demographic and football-related factors, such as race, lineman status, age, months between survey completion, and years since play, did not predict any change in CSS scores over the time between the two assessments. Importantly, we also did not see evidence that changes in pain, cognition, overall health, depression, and anxiety were associated with ΔCSS score. If anything, the CSS score was lower for those who reported health conditions that got worse, although these were far from significant.

Our results suggest that the CSS scale shows good test–retest reliability for measuring concussion history, and that change in current health status is not associated with changes in reporting on the CSS scale. If anything, worse health was related to reduce CSS reporting. This is an important finding given that there can be concern that those who experience more severe outcomes may be more likely to overestimate previous exposures.^40^ This would be differential misclassification, which can produce biased estimates of the relationship between the exposure and the outcome.^40^ For example, currently, public media presentations have linked head injury and later life cognitive health outcomes among former professional ASF players.^23–25^ As a result, there is concern that participants experiencing impaired subjective cognition may overreport previous head injury. This could impact prior studies showing CSS associations with key health variables important for long-term health, such as depression,^4^ anxiety,^4^ self-reported cognitive dysfunction,^4^ endocrine dysfunction^1^ and cardiometabolic outcomes,^2,3^ in which case the associations seen could be the result of reverse causation rather than the head injury history causing the health outcome. Our current findings that change in health conditions were not associated with change in CSS reporting suggest that reverse causation is not occurring, and that the CSS appears to be a consistent retrospective head injury assessment tool with reporting not influenced by current health status.

The OSU TBI-ID is the only other retrospective scale that measures lifetime exposure to traumatic brain injury.^15^ That survey is interview-based and to the best of our knowledge, has not been tested as a self-administered survey,^16,17,41^ although there is a modified version, in which the survey is administered by an interviewer over the phone. Test–retest reliability of the telephone-administered OSU TBI-ID was tested over an interval of up to 15 months.^17^ Among cumulative measures, only a number of TBIs with LOC of 30 min or more showed good test–retest reliability (ICC = 0.7), while all other cumulative indices had ICCs ≤ 0.21. Indices of severity had reasonable reliability, but these are not measures that the CSS collects. One other report found that the number of self-reported LOC occurrences among 27 former NFL players agreed perfectly when asked again 1–2 years later, and there was also reasonable agreement for the reported number of concussions.^13^ Our results with the CSS scale show similar reliability to assess previous concussion history as the OSU-TBI, but over an even longer average time interval of over 6 years.

A number of limitations should be noted. First, former ASF players who participated in this study may not be representative of the larger population of former players. However, this cohort has previously been shown to have similar distributions of responses across age, years played, and positions played compared to the entire potential cohort of former players who have not yet participated.^27^ Furthermore, the number of participants who completed questions on current health status is relatively small and may also limit the generalizability of the results. However, we did find this cohort of players to be relatively similar demographically to the larger cohort, although the convenience sample was on average older than the larger cohort (Supplementary Table S1). Finally, due to the nature of concussion, there is no objective method to validate one’s exposure to head injury. While the most comparable benchmark to a gold standard is a clinical diagnosis of concussion or TBI, clinical diagnoses contain a certain level of subjectivity and ambiguity owing to the nonspecific nature of some CSS, and can be particularly suspect in the context of something like football where there are strong incentives to under report in the moment (e.g., to be able to return to play). Although it is possible heightened attention from the media on concussion during sport may have sensitized former athletes to the significance of concussion and altered their recall of the injuries they sustained during their professional career, the results of this study suggest that current health conditions are not affecting CSS reporting and that the CSS shows good test–retest reliability in concussion history recall even an average of several years later and many years since professional play. Given the interest in the long-term effects of prior head injury exposure on long-term health exposures, it is important that sports medicine and occupational exposure scientists develop, evaluate, and improve upon retrospective head injury assessment tools. Additional research that administers the CSS to active athlete populations (who show increased cardiovascular risks after concussion; Rhim et al.) could potentially identify adverse outcomes that occur relatively recently after head injury or sports season.

In conclusion, the CSS scale exhibited very good test–retest reliability amongst a population of former American-style professional football players. To the best of our knowledge, this is one of the only reliability studies of a remote self-administered retrospective head injury assessment instrument. As research aimed at identifying and protecting former players from adverse outcomes associated with head injury progresses, there is an increased need for reliable tools measuring concussion history that are not influenced by current health status. Future studies of other brain-injured populations (e.g., former military personnel, other contact sport athletes) may consider remotely administering the CSS scale, especially for geographically diverse populations.

## Transparency, Rigor, and Reproducibility

This study was approved by the Institutional Review Board at the Harvard T.H. Chan School of Public Health. This retrospective study qualifies as human subject research. A total of 335 participants were included in this study. The 10-item CSS scale was developed by the Football Player’s Health Study at Harvard University to assess head injuries acquired while actively playing football. Participants were assigned unique study IDs to protect participant names from being associated with any identifying or health information. The datasets presented in this article are not readily available because participant survey responses used in this study could be used to recognize the identities of participants. The data are under the protection of a Certificate of Confidentiality granted by the NIH. The methods section explains the nature of the statistical tests used. All analyses were performed by N.A.K., using R Language for Statistical Computing.^39^

## Abbreviations Used

ASF: American-style football
CSS: Concussion signs and symptoms
FPHS: Football Players Health Study
LOC: Loss of consciousness
NFL: National Football League
OSU TBI-ID: Ohio State University Traumatic Brain Injury Identification Method
Neuro-QOL: Quality of Life in Neurological Disorders
TBI: Traumatic brain injury
