# Supplementary material for: Reliability of Concussion Signs and Symptoms Reporting Among Former Professional American-Style Football Players
Source: Neurotrauma Rep. 2025 Jul 22;6(1):578–85. doi: 10.1177/08977151251362274 (PMC12413250; doi:10.1177/08977151251362274)
Supplement: Supplementary Tables [file 08977151251362274_supplementary_tables.docx]

**Supplemental Material**

**eTable 1:** Demographics of the larger cohort of participants who completed the concussion signs and symptoms scale only at time 1 (total cohort), and the sample of participants who were included in the analysis, stratified by repeated measures and convenience sample.

|  | **Total cohort** | **Analytical sample** | |
| --- | --- | --- | --- |
|  | Overall (N=4189) | Repeated Measures (N=335) | Convenience Sample (N=81) |
| **Age, mean (SD)** | 51.8 (14.4) | 52.1 (14.2) | 59.0 (13.2) |
| **Race** |  |  |  |
| Black | 1634 (39.0%) | 115 (34.3%) | 34 (42.0%) |
| White | 2376 (56.7%) | 205 (61.2%) | 44 (54.3%) |
| Other | 126 (3.0%) | 9 (2.7%) | 2 (2.5%) |
| Missing | 53 (1.3%) | 6 (1.8%) | 1 (1.2%) |
| **Years since play, mean (SD)** | 23.6 (14.3) | 24.5 (13.7) | 30.4 (13.4) |
| N-Miss | 5 | 0 | 0 |
| **Lineman Status** |  |  |  |
| Yes | 1420 (33.9%) | 119 (35.5%) | 29 (35.8%) |

**eTable 2:** Demographics of the analytical sample compared to those excluded from analysis. Participants were excluded if missing at least one symptom response.

|  | Analytical sample (N=335) | Excluded from Analysis (N=77) |
| --- | --- | --- |
| **Age, mean (SD)** | 52.1 (14.2) | 56.2 (12.8) |
| **Race** |  |  |
| Black | 115 (34.3%) | 24 (31.2%) |
| White | 205 (61.2%) | 50 (64.9%) |
| Other | 9 (2.7%) | 1 (1.3%) |
| Missing | 6 (1.8%) | 2 (2.6%) |
| **Years Since Play, mean (SD)** | 24.5 (13.7) | 28.1 (12.2) |
| **Lineman Status** |  |  |
| Yes | 119 (35.5%) | 21 (27.3%) |
| **Months Between Survey Completion, mean (SD)** | 74.5 (41.2) | 3.1 (6.0) |
| N-Miss | 3 | 0 |
| **CSS score at time 1, mean (SD)** | 30.2 (25.5) | 30.7 (27.3) |
| N-Miss | 0 | 41 |
| **CSS score at time 2, mean (SD)** | 29.1 (25.2) | 30.8 (29.7) |
| N-Miss | 0 | 48 |
| **CSS score difference, mean (SD)** | -1.1 (19.8) | - |
| N-Miss | 0 | 77 |

Note: CSS=Concussion signs and symptoms scale

**eTable 3:** Demographics and health outcomes for the convenience sample participants who completed the concussion signs and symptoms scale and health information at two timepoints.

|  | Convenience Sample (N=81) |
| --- | --- |
| **Age, mean (SD)** | 59.0 (13.2) |
| **Race** |  |
| Black | 34 (42.0%) |
| White | 44 (54.3%) |
| Other | 2 (2.5%) |
| Missing | 1 (1.2%) |
| **Years Since Play, mean (SD)** | 30.4 (13.4) |
| **Lineman Status** |  |
| Yes | 29 (35.8%) |
| **Months Between Survey Completion, mean (SD)** | 7.8 (12.1) |
| N-Miss | 0 |
| **CSS score at time 1, mean (SD)** | 31.5 (28.3) |
| **CSS score at time 2, mean (SD)** | 31.7 (28.3) |
| **CSS score difference, mean (SD)** | 0.2 (12.3) |
| **Pain at time 1, mean (SD)** | 4.7 (2.5) |
| **Pain at time 2, mean (SD)** | 4.6 (2.5) |
| **ΔPain, mean (SD)** | -0.1 (1.1) |
| **Perceived cognitive difficulties at time 1, mean (SD)** | 40.3 (9.8) |
| **Perceived cognitive difficulties at time 2, mean (SD)** | 39.7 (9.4) |
| **ΔNeuro-QOL, Mean (SD)** | 0.6 (2.4) |
| **Poor general health at time 1, mean (SD)** | 2.8 (0.9) |
| **Poor general health at time 2, mean (SD)** | 2.9 (0.8) |
| N-Miss | 1 |
| **ΔHealth, mean (SD)** | 0.0 (0.4) |
| N-Miss | 1 |
| **Depression score at time 1, mean (SD)** | 1.4 (1.8) |
| **Depression score at time 2, mean (SD)** | 1.4 (1.7) |
| **ΔDep, mean (SD)** | 0.0 (0.9) |
| **Anxiety score at time 1, mean (SD)** | 1.6 (1.8) |
| **Anxiety score at time 2, mean (SD)** | 1.6 (1.7) |
| **ΔAnx, mean (SD)** | 0.0 (0.9) |

Note: CSS=Concussion signs and symptoms scale
